# Elementary Process for CVD Graphene on Cu(110): Size-selective Carbon Clusters

**DOI:** 10.1038/srep04431

**Published:** 2014-03-21

**Authors:** Jialin Zhang, Zhunzhun Wang, Tianchao Niu, Shengnan Wang, Zhenyu Li, Wei Chen

**Affiliations:** 1 Department of Physics, National University of Singapore, 2 Science Drive 3, 117542, Singapore; 2 Hefei National Laboratory for Physical Sciences at Microscale, University of Science and Technology of China, Hefei 230026, China; 3 Department of Chemistry, National University of Singapore, 3 Science Drive 3, 117543, Singapore; 4 These authors contributed equally to this work.

## Abstract

Revealing the graphene growth mechanism at the atomic-scale is of great importance for achieving high quality graphene. However, the lack of direct experimental observation and density functional theory (DFT) verification hinders a comprehensive understanding of the structure of the carbon clusters and evolution of the graphene growth on surface. Here, we report an in-situ low-temperature scanning tunneling microscopy (LT-STM) study of the elementary process of chemical vapor deposition (CVD) graphene growth via thermal decomposition of methane on Cu(110), including the formation of monodispersed carbon clusters at the initial stage, the graphene nucleation and the ripening of graphene islands to form continuous graphene film. STM measurement, supported by DFT calculations, suggests that the carbon clusters on the surface are C_2_H_5_. It is found that graphene layers can be joined by different domains, with a relative misorientation of 30°. These graphene layers can be decoupled from Cu(110) through low temperature thermal cycling.

Ever since its mechanical exfoliation from small mesas of highly oriented pyrolytic graphite, graphene has spurred a tremendous of interest because of its exceptional electronic and mechanical properties, such as anomalous quantum Hall Effect (QHE), long-range ballistic transport, high carrier mobility, tunable band gap, high elasticity and intrinsic strength^1^,^2^,^3^,^4^. All these qualify graphene as a promising material for applications in microelectronic and spintronic devices^1^,^2^,^3^, sensors^5^, supercapacitors^6^, building blocks for multifunctional composites^7^ as well as for structural and mechanical applications^4^. Motivated by these extraordinary properties and numerous potential applications, a number of graphene fabrication methods have been explored, including the micromechanical cleavage of graphite^1^, thermal decomposition of SiC^8^, reduction of chemically functionalized graphene^9^,^10^,^11^,^12^, chemical exfoliation of graphite^13^ and transition metal (TM)-catalyzed chemical vapor deposition (CVD)^14^,^15^,^16^,^17^,^18^,^19^,^20^,^21^,^22^,^23^,^24^,^25^,^26^, and so on. Graphene prepared by cleavage and exfoliation of graphite shows superior transport properties, but its size is usually limited to micrometers and the productivity of this method is very low^1^. Epitaxial graphene on SiC allows larger area synthesis^27^, but this method induces noticeable densities of defects and achieving large graphene domains with uniform thickness remains a challenge^28^. Transition metal assisted growth of graphene, which provides many unique advantages, such as industrial scalability^21^, relatively low temperature processing^18^, easy transferring onto other substrates^23^, has received the most attention.

The graphene growth on TM surfaces is based on high-temperature pyrolysis of hydrocarbons and different growth mechanisms can be involved according to the carbon solubility limit in the metal. For the growth on TM where carbon is soluble, the graphene forms when the sample is cooled and carbon segregates on the surface (surface segregation)^29^,^30^,^31^,^32^; but for TM with very low carbon solubility, the synthesis is limited to the surface of the catalyst and mainly involves surface diffusion and nucleation of carbon atoms. The growth of graphene can be accomplished by CVD via two approaches: directly cracking the carbon source on TM surface at a high temperature or temperature programmed growth (TPG) via room temperature adsorption of the molecules followed by pyrolysis and graphene growth at a fixed elevated temperature^17^.

Graphene growth has been demonstrated on a variety of TMs. For example, Li and colleagues reported a CVD method that used copper-foil to produce single crystal graphene with dimensions of up to 0.5 mm^20^; Bae and col leagues demonstrated a roll-to roll production of 30 inch graphene films for transport electrodes^21^; Gao and colleagues showed the repeated growth and bubbling transfer of graphene with millimeter-size single-crystal grains using platinum^24^. Additionally, intensive theoretical efforts have been devoted to revealing the growth mechanism^14^,^33^,^34^,^35^,^36^,^37^,^38^,^39^,^40^,^41^,^42^,^43^. Using first-principle calculations, Chen et al. found on flat surfaces of Ir(111) and Ru(0001), two carbon atoms repel each other; while they prefer to form a dimer on Cu(111)^37^. Zhang et al. also revealed that C_2_H_2_ can be easily formed on a Cu(111) surface, which represents a more favorable reaction path compared to CH dissociation^38^. By careful optimization of the supported carbon clusters C_N_ on Ni(111), Gao et al. indicated a ground state structure transition from a one-dimensional (1D) carbon chain to a two-dimensional (2D) sp^2^ carbon network at N ~ 10–12^40^; while Wesep and co-workers proposed an energetic preference for the formation of stable 1D carbon nanoarches consisting of 3–13 atoms on Cu(111) surface^43^. Explored by ab initio calculations, Yuan et al. showed that the core-shell C_21_ is a very stable magic carbon cluster on Rh(111), Ru(0001), Ni(111) and Cu(111) surfaces^42^. Zangwill et al. predicted that an immobile island composed of six five-atom carbon clusters as the smallest stable precursor to graphene growth on metals^41^. Despite these inspiring achievements, most of these theoretical studies only address the number of carbon atoms, and the precise determination of hydrogen atoms within the cluster is rare. Moreover, very little of the growth mechanism in the initial nucleation stages of carbon atoms has been revealed experimentally^15^,^16^,^44^. In this regard, atomic-scale characterization of a complete process of graphene growth in combination with theoretical calculations is of great importance, for both fundamental interest and achieving high quality graphene.

Here, we report an atomic scale characterization of the elementary process of CVD graphene growth via thermal decomposition of methane (CH_4_) on Cu(110) using low-temperature scanning tunneling microscopy (LT-STM), including the formation of monodispersed carbon clusters at low temperature, nucleation and ripening of graphene islands at high temperature. Combined with first principles calculations, the monodispersed carbon clusters are identified as C_2_H_5_. Different domains stitch together to form a graphene layer, with a preference angle of 30° at the grain boundaries. These graphene layers can be decoupled from Cu(110) through low temperature thermal cycling.

## Results

**Figure 1:**
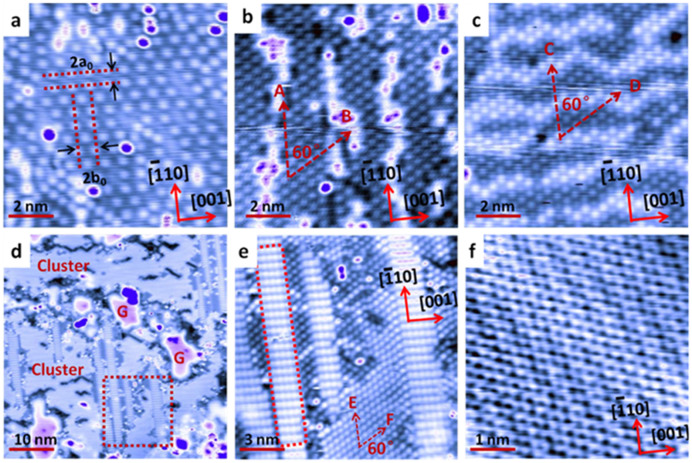
Evolution of carbon clusters and formation of grapheme on Cu(110). (a) STM image (V_tip_ = 0.25 V, 10 × 10 nm^2^) of the low coverage carbon clusters on Cu(110), which were formed upon the deposition of CH_4_ at room temperature and subsequent annealing at 480°C in CH_4_ at a pressure of 2 × 10^−5^ mbar for 50 min. (b) STM image (V_tip_ = 1 V, 10 × 10 nm^2^) of carbon clusters at higher coverage; A and B indicate the direction of the unit cell vectors. (c) Hexagonally close packed cluster structure formed by further increasing the carbon clusters coverage. (V_tip_ = 0.2 V, 10 × 10 nm^2^), C and D indicate the direction of the unit cell vectors. (d) Large scale STM image (V_tip_ = 0.5 V, 50 × 50 nm^2^) of carbon clusters and small graphene flakes on Cu(110) by annealing Cu(110) in CH_4_ at 550°C at a pressure of 2 × 10^−5^ mbar for 130 min, where the graphene flakes are indicated by “G”. (e) The corresponding high resolution STM image (V_tip_ = 0.1 V, 15 × 15 nm^2^) showing the hexagonally close packed carbon clusters in panel 1 (d), where E and F indicate the direction of the unit cell vectors. (f) The atomically resolved STM image (V_tip_ = 0.03 V, 5 × 5 nm^2^) showing the 1 × 1 graphene lattice.

As shown by the high magnification STM image in Fig. 1a, upon the deposition of CH_4_ at room temperature (RT) and subsequent annealing at 480°C in CH_4_ at a pressure of 2 × 10^−5^ mbar for 50 min, the Cu(110) surface was almost decorated with carbon clusters of monodispersed size. Each carbon cluster appears as a bright spot with an identical size of 0.4 nm. Careful inspection of the STM image reveals that the surface is decorated by isolated but well-defined superstructures, where the carbon clusters are adsorbed in an epitaxial relationship with the underling Cu(110). As indicated by the dashed lines in Fig. 1a, the minimum distance between two neighboring row is 2a_0_ of 0.512 nm; while it is 2b_0_ of 0.723 nm between two columns (a_0_ and b_0_ are the unit cell dimensions of Cu(110)). It can also be revealed that the carbon cluster arrays are aligned precisely with the crystal orientation of the underlying Cu(110). The carbon cluster at this low coverage was referred to as “cluster 1” with a density around 2.70 × 10^14^/cm^2^. Previous theoretical studies proposed that carbon dimmers are energetically favorable on the Cu surface^37^,^38^,^39^. Therefore, we tentatively assign these carbon clusters as carbon dimers (C_2_H_x_).

Further increasing the coverage of the carbon clusters can result in the formation of a hexagonally close packed structure, as shown in Fig. 1b. The coverage of the carbon clusters can be increased through low temperature thermal cycling as described in the supporting information. Some gaps can still be observed between the ordered domains. However, the carbon clusters in each ordered domain posses the unit cell with a = 0.515 nm, b = 0.500 nm and an inclusion angle of 60°, as indicated by arrows A and B. Upon saturation of the carbon clusters on the surface, they formed highly ordered close packed structure over the surface, as shown in Fig. 1c. The unit cell was further reduced to c = 0.450 nm, d = 0.480 nm with an unchanged inclusion angle of 60°. At this stage, the carbon cluster density was increased to 10.9 × 10^14^/cm^2^, referred to as “cluster 2”. In this regime, the arrangement is supposed to be cluster-cluster interaction dominated. Some brighter lines can be frequently observed, induced by the stress relaxation at high cluster coverage with increased lateral inter-cluster interaction.

Annealing the Cu(110) surface at high temperature at 550°C in CH_4_ at a pressure of 2 × 10^−5^ mbar for 130 min can promote the nucleation of small graphene flakes. As shown in Fig. 1d, at this stage the carbon clusters co-exist with the small graphene flakes which are indicated as “G”. The high magnification STM image in Fig. 1e reveals that the clusters on Cu(110) are “cluster 2”. The directions of the unit cell are indicated by arrows E and F, with lateral dimensions of e = 0.450 nm, f = 0.480 nm and an inclusion angle of 60°. The bright stripes inserted between these clusters are clean Cu(110) surface but with a 1 × 2 superstructure as highlighted by the red dotted line in Fig. 1e. Figure 1f shows the atomically resolved STM image of the 1 × 1 graphene lattice, and the crystal orientation of the underlying Cu(110) is indicated in the lower right corner.

To obtain the atomic structure of the carbon clusters, the adsorption of various carbon clusters on Cu(110) were simulated using DFT. First, the stability of C_1_H_x_ (0 ~ 4) and C_2_H_x_ (0 ~ 6) clusters on Cu (110) were studied. We define the formation energy in equation (1)

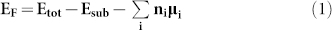
where E_tot_ is the total energy of the adsorbed system, E_sub_ is the energy of clean Cu (110) substrate, μ_i_ and n_i_ (i = C, H) represent chemical potential and the number of atoms in the cluster, respectively. Considering the equilibrium of CH_4_ and H_2_, the relationship of μ_H_ and μ_C_ in unit of electron volt can be obtained as equation (2) by the process described in the supporting information: 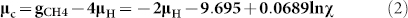
Here, χ is the ratio of the partial pressures of CH_4_ and H_2_.

**Figure 2:**
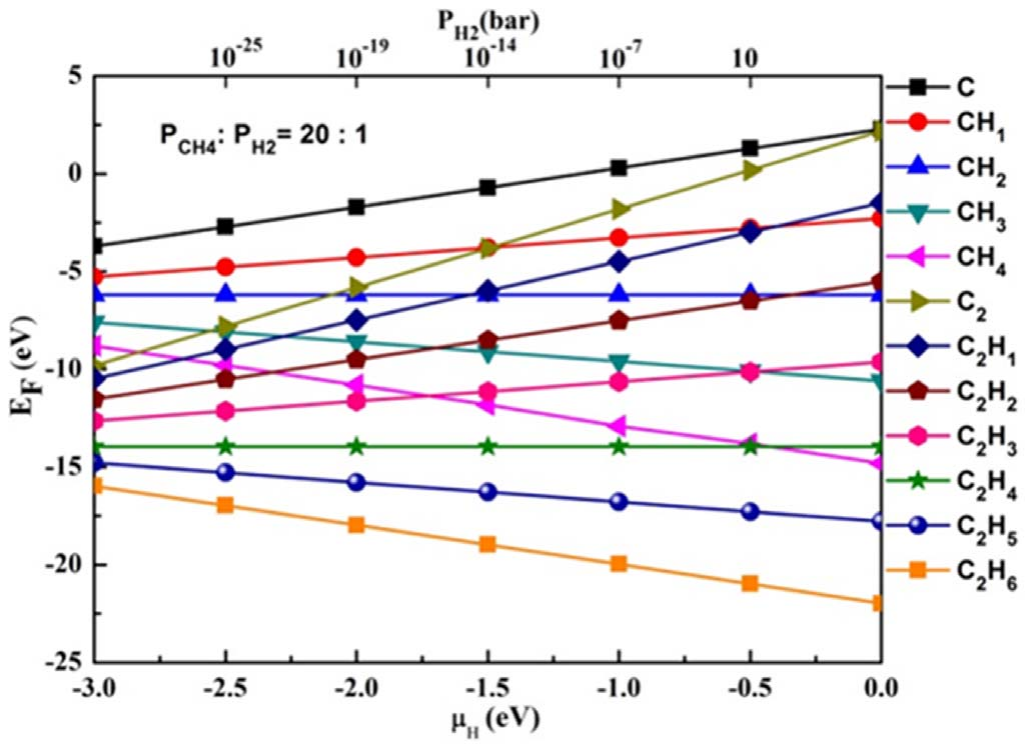
Relationship of formation energy and chemical potential of H or the pressure of H_2_. Relationship of adsorption energy and chemical potential of H or the pressure of H_2_ during CVD growth of carbon-clusters on Cu (110) at T = 527°C. The ratio of partial pressures of CH_4_ and H_2_ is χ = 20.

For each carbon cluster species, the most stable adsorption configuration was found by checking different adsorption sites on Cu(110) surface, including the hollow site (H-site), bridge-long site (B_long_ site), bridge-short site (B_short_ site) and Top site (T-site)^45^. Figure 2 shows the formation energy of various carbon cluster species as a function of the chemical potential of H (thus the partial pressure of H_2_). The χ here was set to be 20:1; we also tested χ = 1:20, which gave similar results.

From Fig. 2, it is easy to find that clusters C_2_H_6_ and C_2_H_5_ are the two most stable species under all physical H_2_ partial pressure. Although the formation energy of C_2_H_6_ is very large, as a close shell molecule, its adsorption energy is expected to be very small, and it's hence easy to desorb from Cu(110) at high temperature. The average lifetime of C_2_H_6_ and C_2_H_5_ can be estimated by their adsorption energy E_a_ via 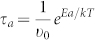 46. According to our calculations, adsorption energy of C_2_H_6_ and C_2_H_5_ on Cu(110) surface are 0.41 and 2.85 eV, respectively. υ_0_ is about 10^13^ s^−1^. Therefore, their average lifetime on the surface at 480°C is 5.5 × 10^−11^ and 1.2 × 10^6^ s, respectively. Such a short lifetime makes C_2_H_6_ not be able to be observed by STM. Therefore, C_2_H_5_ could be the most possible abundant species from the thermodynamic point of view.

**Figure 3:**
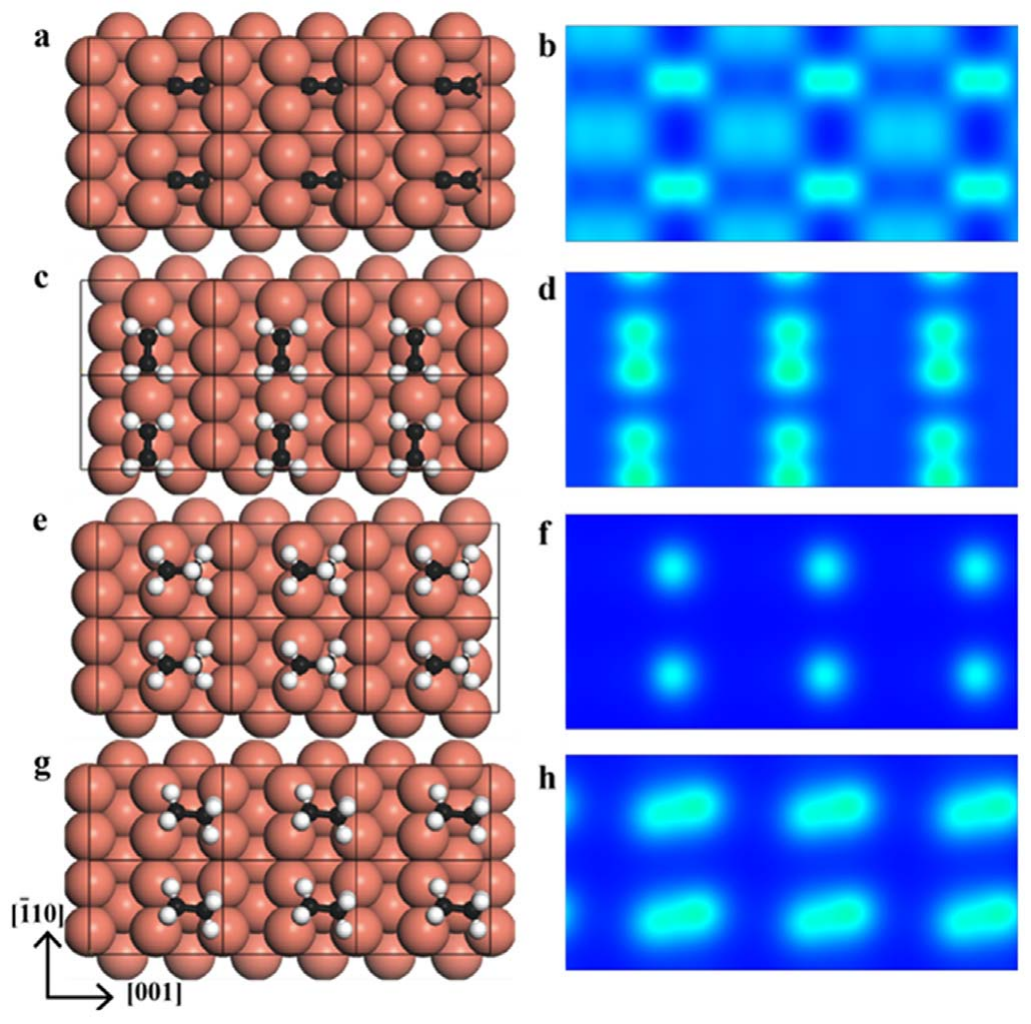
Optimized structures (left panels) and simulated STM images. Optimized structures (left panels) and simulated STM images (right panels) of (a, b) C_2_, (c, d) C_2_H_4_, (e, f) C_2_H_5_, and (g, h) C_2_H_6_. The integrated density of states from 0.25 V below E_F_ to the Fermi level is used to simulate the STM image, which represents the HOMO of the carbon clusters.

STM images of several partially dehydrogenated carbon dimer species were also simulated using the Tersoff and Hamann approximation^47^. Figure 3 shows the optimized structures and simulated STM images of C_2_, C_2_H_4_, C_2_H_5_ and C_2_H_6_. The optimized unit cell of the carbon cluster is 2a_0_ = 0.504 nm, 2b_0_ = 0.713 nm. Among these carbon clusters, the simulated STM image of C_2_H_5_ is in good agreement with the experimental results. All other stable species cannot reproduce the experimental circular shape. Hence, the basic structures of the carbon clusters are elucidated by the STM images in combination with DFT calculations as C_2_H_5_.

**Figure 4:**
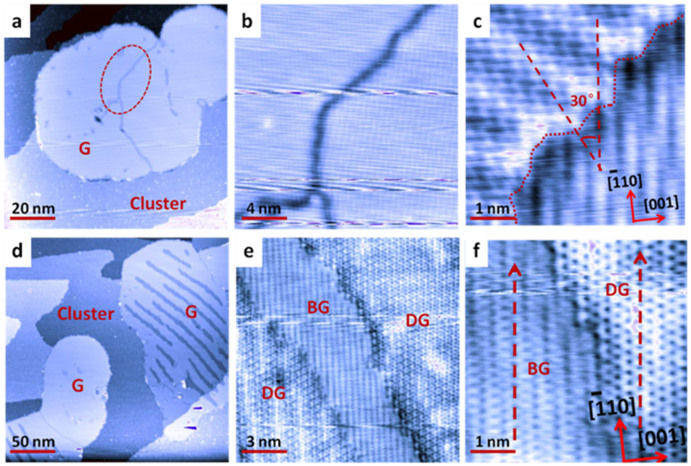
STM images showing the jointed domains and low temperature thermal cycling induced decoupling of graphene from Cu(110) substrate. (a) Large scale STM image (V_tip_ = 1 V, 100 × 100 nm^2^) showing one graphene flake jointed by different domains, which was formed by low temperature thermal cycling and subsequent annealing of the carbon clusters on Cu(110) up to 720°C. (b) The corresponding high resolution STM image (V_tip_ = 0.04 V, 20 × 20 nm^2^) showing the domain boundary. (c) The atomically resolved STM image (V_tip_ = 0.03 V, 5 × 5 nm^2^) illustrating two distinct graphene orientations, the upper domain is orientated at an angle of 30° relative to the lower domain. (d) Large scale STM image (V_tip_ = 1.0 V, 250 × 250 nm^2^) of Cu(110) covered by large flakes of graphene and carbon clusters. (e) A continuous single layer of graphene with different contrast (V_tip_ = 0.05 V, 15 × 15 nm^2^). (f) (V_tip_ = −0.01 V, 5 × 5 nm^2^) The corresponding high resolution STM images of panel (e), where the orientation of graphene is indicated by the red arrows.

Large graphene flakes can be achieved through low temperature thermal cycling process as described in the supporting information. Figure 4a shows a large scale STM image of a flake of graphene film on Cu(110) interconnected by two graphene grains, forming a grain boundary in between as indicated by the red ellipse. Close-up (Fig. 4b) and the corresponding atomic-resolution STM images (Fig. 4c) reveal that the two graphene grains are stitched together to form a continuous film with a relative misorientation of 30°. The detailed atomic structure at the grain boundary cannot be identified from our STM image, but it has been theoretically proposed and experimentally conformed as a series of pentagons, heptagons and distorted hexagons^25^,^48^. The graphene grows in different orientations with respect to the underlying lattice, resulting in two different moiré patterns. As shown in Fig. 4c, the lower right panel shows a moiré superstructure almost aligned with the underlying Cu(110) lattice, referred to as R0 phase. The graphene lattice of the upper left panel shows a different moiré pattern with a larger periodic modulation and is rotated by 30° from the lower R0 phase, referred to as R30 phase. Supplementary Fig. S3 on line shows a graphene film joined by multi-domains taken from a different location on Cu(110), which also shows a 30° misorientation. The preference of around 30° misorientation between two domains has also been reported by other groups^19^,^25^. For graphene grown on Ru(0001), only one orientation can be observed, due to the strong interaction between graphene and Ru^29^. The two dominating orientations observed here and the fact that graphene can grow continuously across Cu step edges could indicate a weaker graphene-Cu interaction when compared with Ru.

As described in the supporting information, during the experiment, we introduced the low temperature thermal cycling method to increase the carbon cluster coverage. Figure 4d shows the STM image of large flakes of graphene coexisting with carbon clusters on Cu(110). After repeating several cycles of low temperature thermal cycling, the graphene flakes on the surface possess two stripe-shaped contrasts. Comparison between Supplementary Fig. S2 on line and Fig. 4d reveals that the appearance of those bright stripes are same with the previous small graphene islands; while the dark stripes are newly produced during the low temperature thermal cycling. Close up STM image in Supplementary Fig. S4 on line and Fig. 4f reveals that the bright and dark stripes alternated between each other with a continuous boundary. As shown in Fig. 4e, the bright stripes (BG) show moiré pattern resembling the underlying Cu(110); while the dark stripes (DG) display prefect hexagonal graphene lattice. These contrasts result from the modulation by different interactions with the underlying Cu(110). The appearance of the prefect hexagonal graphene lattice in DG suggests that the graphene in this region is physically decoupled from the underlying Cu(110).

The formation of such physically decoupled graphene can arise from the intercalation at the graphene/Cu(110) interface by hydrogen atoms released from CH_4_ decomposition, similar to the previously reported hydrogen^49^, lithium^50^, oxygen^51^, and fluorine intercalation to form quasi-free-standing graphene^52^; or from the strain relief during the annealing/cooling cycles due to the different thermal expansion of graphene film and Cu substrate^53^. More controlled experiment and detailed theoretical calculations will be carried out to unravel the decoupling mechanism.

## Discussion

Through the combination of the LT-STM and DFT calculations, we reveal the elementary process of graphene growth on Cu(110) surface via thermal decomposition of CH_4_. Low temperature annealing (>480°C) in CH_4_ results in the formation of carbon clusters at the initial stage; further high temperature annealing (>550°C) activates the graphene nucleation; prolonged annealing in the absence of CH_4_ propels the diffusing and ripening of these graphene island to form continuous graphene films extended over the surface. Low temperature thermal cycling induced decoupling of graphene from Cu(110) has also been demonstrated. Our systematic investigations identify the fundamental carbidic building blocks by STM measurement, and further elucidate their atomic structures through DFT calculations. Our work could lay the foundation for providing rational design rules for synthesis of large area single crystalline graphene films.

## Methods

### Growth of graphene on Cu(110)

Graphene was grown on a single crystal Cu(110) via thermal decomposition of CH_4_. Prior to the deposition of CH_4_, Cu(110) substrate was cleaned by a few cycles of Ar^+^ ion bombardment and subsequent annealing at 530°C. The CH_4_ gas was introduced into the growth chamber through a leak valve, and the pressure was monitored by a cold cathode gauge. A typical growth procedure is as follows: the Cu(110) substrate was exposed to CH_4_ at a pressure of 2 × 10^−5^ mbar for 20 min; annealing the sample at 480°C in CH_4_ at a pressure of 2 × 10^−5^ mbar resulted in the formation of carbon clusters; further annealing the sample in the absence of CH_4_ at 550°C initiated the graphene nucleation; prolonged annealing without CH_4_ at higher temperature up to 720°C propelled the ripening of graphene islands.

### Characterization of graphene in UHV LT-STM

The LT-STM experiments were carried out in a custom-built multichamber ultra-high-vacuum (UHV) system with base pressure better than 1.0 × 10^−10^ mbar, housing an omicron LT-STM interfaced to a Nanonis controller. All STM imaging were performed at 77 K using constant current mode with an electrochemically etched tungsten tip. All the bias voltage was applied to the tip^54^.

### Structural models of clusters on Cu (110) surface

Stability of C_1_H_x_ (0 ~ 4) or C_2_H_x_ (0 ~ 6) clusters on Cu (110) surface were studied using DFT calculations. A 5-layer slab with a 20 Å vacuum layer was used as the substrate. The bottom layer was fixed to its bulk configuration and all other atoms were fully relaxed. A (3 × 4) supercell was chosen to make sure that clusters were separated to their neighboring clusters by more than 10 Å. In STM simulation, a (2 × 2) supercell was chosen according to the experimental coverage.

### Calculation details

All the calculations were performed using DFT implemented in the Vienna Ab Initio Simulation Package (VASP) within the generalized gradient approximation^55^,^56^ plus DFT-D2 van der Waals (vdW) correction^57^. The exchange-correlation functional of Perdew-Burke-Ernzerhof^58^ and the projector-augmented wave^59^ methods were used. The plane-wave basis cutoff energy was set to 500 eV. The criteria of convergence for energy and force were set to 10^−5^ eV and 0.02 eV/Å. For the (3 × 4) and (2 × 2) models, (7 × 7 × 1) and (10 × 14 × 1) k-point grids were used, respectively. STM images were simulated using the Tersoff and Hamann approximation^47^. The lattice parameter of bulk Cu was optimized to be 3.564 Å^60^.

## Author Contributions

W.C. conceived and designed the experiments; J.L.Z. and T.C.N. performed the experiments; Z.Z.W., S.N.W. and Z.Y.L. performed theoretical calculations; W.C. and J.L.Z. wrote the manuscript. All authors contributed to writing and revising the manuscript.

## Supplementary Material

Supplementary Information Supplementary Information
