# Supplementary material for: Elementary Process for CVD Graphene on Cu(110): Size-selective Carbon Clusters
Source: Sci Rep. 2014 Mar 21;4:4431. doi: 10.1038/srep04431 (PMC3961735; doi:10.1038/srep04431)
Supplement: Supplementary Information [file srep04431-s1.pdf]

Supplementary information

Elementary Process for CVD Graphene on Cu(110): Size-selective Carbon Clusters

Jialin Zhang[1,#], Zhunzhun Wang[2,#], Tianchao Niu[3], Shengnan Wang[2], Zhenyu Li[2,*], Wei Chen[1,3,*]

[1]Department of Physics, National University of Singapore, 2 Science Drive 3, 117542, Singapore

[2]Hefei National Laboratory for Physical Sciences at Microscale, University of Science and Technology of China, Hefei 230026, China

[3]Department of Chemistry, National University of Singapore, 3 Science Drive 3, 117543, Singapore

[#] Both authors contributed equally to this work.

1. Deviation of the relationship between μ_H and μ_C

μ_i and n_i (i= C, H) represent chemical potential and the number of atoms in the cluster, respectively. Considering the equilibrium of CH_4 and H_2, the relationship of μ_H and μ_C in units of electronvolt can be obtained by following process[1].

Define the reference pressure P_0 = 1bar and according to the NIST-JANAF thermochemical tables[2], at 530 ^{o}C, we have

$$\mu_H(T,P_0) = \frac{1}{2}[h(T,P_0) - h(0,P_0) - TS(T,P_0)]$$
$$= -0.541 \quad (1)$$

Then, under arbitrary pressure, we have

$$\mu_H(T,P) = \mu_H(T,P_0) + \frac{1}{2}k_B T \ln\frac{P_{H_2}}{P_0}$$
$$= -0.541 + 0.0345\ln\frac{P_{H_2}}{P_0} \quad (2)$$

For methane, we have

$$\Delta g_{CH_4}(T,P_0) = h(T,P_0) - h(0,P_0) - TS(T,P_0)$$
$$= -1.568 \quad (3)$$

Taking one-half of the H_2 energy and a carbon atom energy as a reference, the DFT-calculated energy of CH_4 is -9.209 eV. So, we have

$$g_{CH_4}(T,P) = -9.209 + \Delta g_{CH_4}(T,P_0) + k_B T \ln \frac{P_{CH_4}}{P_0}$$

$$= -10.777 + 0.0689 \ln \frac{P_{CH_4}}{P_0} \qquad (4)$$

Define the ratio of the partial pressures of CH_4 and H_2 as χ, then we have the following relationship between μ_H and μ_C at the equilibrium of CH_4 and H_2:

$$\mu_C = g_{CH_4} - 4\mu_H = -2\mu_H - 9.695 + 0.0689 \ln \chi \qquad (5)$$

2. Four adsorption sites on Cu(110)

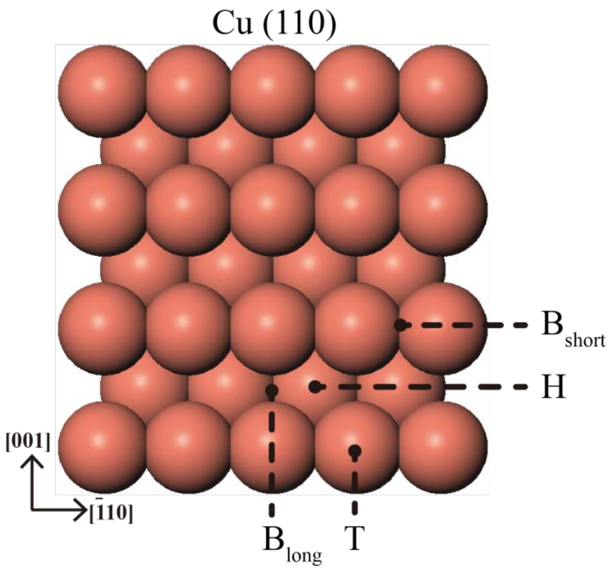

Figure S1 | Four adsorption sites on Cu(110): hollow site (H-site), bridge-long site (B_{long} site), bridge-short site (B_{short} site) and top site (T-site).

3. Graphene size evolution

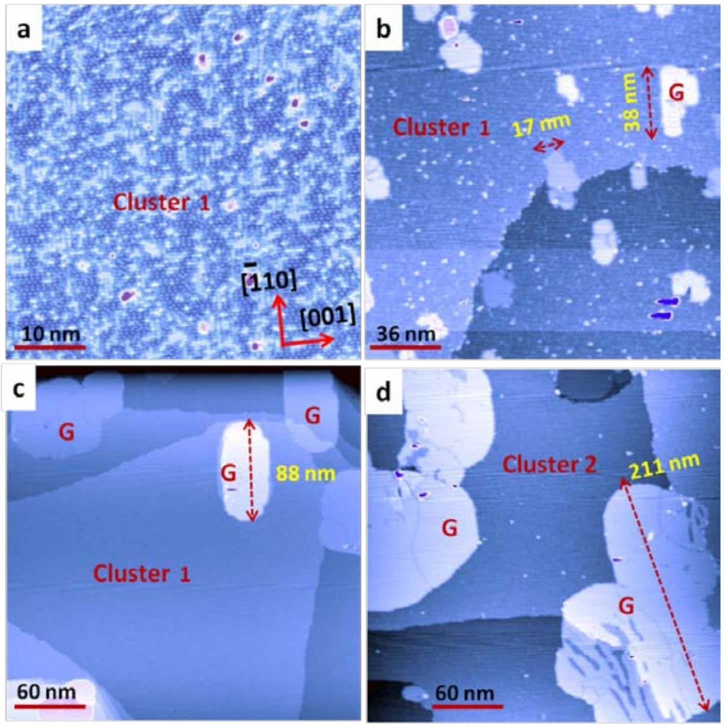

Figure S2 | Evolution of graphene growth via thermal decomposition of CH_4 on Cu(110). a) Cu(110) surface covered by cluster 1 (V_{tip} = 0.5 V, 50 × 50 nm^2). b) STM image (V_{tip} = 1.3 V, 180 × 180 nm^2) of small flakes of graphene formed on the surface by annealing the sample (in panel a) without CH_4 at 600°C for 50 mins. c) Further annealing the sample up to higher temperature 720 °C in the absence of CH_4 can result in the formation of large graphene flakes (V_{tip} = 1 V, 300 × 300 nm^2). d) STM image (V_{tip} = 1.5 V, 300 × 300 nm^2) showing the Cu(110) covered by larger graphene flakes and carbon clusters by repeating the low temperature thermal cycling process2.

Here, we show how to achieve graphene flakes with larger size through low temperature thermal cycling. Figure S2 (a) displays STM images of Cu(110) covered with low coverage “cluster 1”. By annealing these carbon clusters at temperature of 550 °C in the absence of CH_4 for 60 min, the carbon clusters nucleated to form the initial graphene nuclei. Prolonged annealing at higher temperature (600 °C) for 50 min enlarged the size of these nuclei to around several tens of nanometers as shown in Fig. S2 (b). These small graphene islands randomly spread over the terraces or attached to the step edges. We further annealed the sample in the absence of CH_4 up to higher temperature of 720 °C for 3 min. As shown in Fig. S2 (c), this resulted in the

Smoluchowski ripening[3] of the graphene islands by diffusing and stitching to each other. The surface was covered with larger graphene domains with a lower density. The size of the graphene islands is close to hundred nanometers as indicated by the dashed arrow. Further annealing at longer time or higher temperature could not expand the size of graphene domain. As the growth of graphene consumed carbon clusters on the surface and decreased their concentration, the graphene growth stopped once the concentration of carbon clusters was in equilibrium with graphene.

A large super-saturation of carbon cluster is needed to further promote the growth of graphene. This time, a low temperature thermal cycling was adopted to increase the carbon cluster coverage. We first exposed the sample to CH_4 at RT at a pressure of 2×10^{-5} mbar for 20 min, and then annealed the sample in UHV condition at 480 °C for 30 min in the absence of CH_4. Repeating this process for several times, the carbon clusters on the surface can achieve high coverage. Further annealing the sample covered with “cluster 2” up to 670 °C in the absence of CH_4 for 100 min can result in the formation of graphene with larger domain size about 200 nm, as shown in Fig. S2 (d).

4. Characterization of grain boundaries

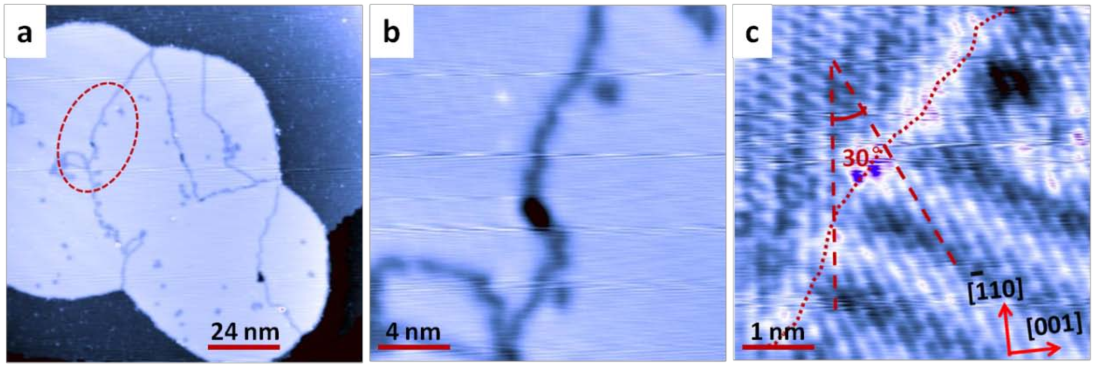

Figure S3 | STM image showing the jointed domains in a different location. a) STM image (V_{tip} = 2 V, 120 × 120 nm^2) of another region showing a continuous graphene flake containing many domains of different orientations. b) The corresponding close-up STM image (V_{tip} = 0.5 V, 20 ×20 nm^2) of the area indicated in panel (a). c) Atomically resolved STM image (V_{tip} = 0.1 mV, 5 × 5 nm^2) revealing two domains connected with a rotation of 30°.

5. Low temperature thermal cycling induced decoupling of graphene from Cu(110)

As afore-mentioned, during the experiment, we introduced the low temperature thermal cycling method to increase both the carbon cluster coverage and the size of graphene flakes. Figure S4 (a) shows the STM image of large flakes of graphene coexisting with carbon clusters on Cu(110). Atomically-resolved STM image in Fig. S4 (b) reveals the boundary between a graphene island and the high coverage carbon clusters. As shown in Fig. S4 (a) and the corresponding close up image in Fig. S4 (c), after low temperature thermal cycling, the graphene flakes on the surface possessed two stripe-shaped contrasts. The bright stripes and dark stripes alternated between each other with a continuous boundary. By increasing the low temperature thermal cycling times, the population of the dark stripes increased, as shown in Fig. S4 (d). At this stage, the surface was covered almost with dark stripes.

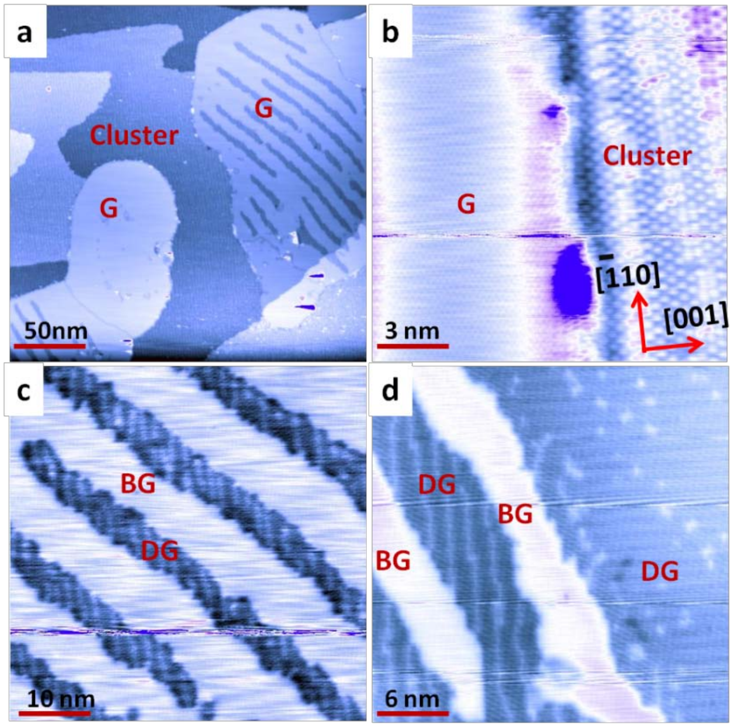

Figure S4 | Low temperature thermal cycling induced decoupling of graphene from Cu(110). a) Large scale STM image (V_{tip} = 1.0 V, 250 × 250 nm^2) of Cu(110) covered by large flakes of graphene and carbon clusters. b) The corresponding high resolution STM image (V_{tip} = 0.02 V, 15 × 15 nm^2) showing the domain boundary between the graphene and the hexagonally close packed carbon clusters. c) Close-up STM image (V_{tip} = 0.5 V, 50 × 50 nm^2) of the graphene flakes with different brightness contrast as indicated in panel (a). d) A continuous single layer of graphene with increased population of dark contrast after additional low temperature theraml cycling (V_{tip} = 2 V, 30 × 30 nm^2).

References

1 Zhang, W., Wu, P., Li, Z. & Yang, J. First-Principles Thermodynamics of Graphene Growth on Cu Surfaces. *J. Phys. Chem. C* **115**, 17782-17787, (2011).

2 Chase, M. W. N. I. o. S. & Technology. *NIST-JANAF thermochemical tables*. (American Chemical Society ; American Institute of Physics for the National Institute of Standards and Technology, 1998).

3 Coraux, J. *et al.* Growth of graphene on Ir(111). *New J. Phys.* **11**, 023006 (2009).
